# Cardiac Manifestations in Post-COVID-19 Multisystem Inflammatory Disease in Children (MIS-C) in Pediatric Patients at a Tertiary Hospital in Bahrain: A Retrospective Study

**DOI:** 10.7759/cureus.83409

**Published:** 2025-05-03

**Authors:** Salsabeel Ebrahim, Wael J Husain, Zakariya Hubail

**Affiliations:** 1 Pediatric Department, Salmaniya Medical Complex, Manama, BHR

**Keywords:** cardiac manifestations, covid 19, kingdom of bahrain, mis-c, pediatric clinical cardiology, post-covid-19, retrospective studies

## Abstract

Background

Children were thought to be immune to the COVID-19 pandemic or have a mild form of the disease. However, reports showed multiple cases of post-COVID-19 inflammatory shock syndrome, later known as the multisystem inflammatory syndrome in children (MIS-C). Although rare, it remains an important cause of morbidity and mortality in pediatric COVID-19 patients.

Objective

To assess the prevalence and characteristics of cardiac manifestations (clinical, radiological, and laboratory findings) of post-COVID-19 MIS-C admitted to the main tertiary hospital in Manama, Bahrain, and to compare such characteristics with regard to age and sex.

Methods

A retrospective study was conducted at a tertiary hospital in Manama City, Bahrain. Data were collected from the hospital's medical records system. All pediatric patients who were admitted during the period from April 2020 to September 2021, with the diagnosis of post-COVID-19 multisystem inflammatory syndrome in children, were included. The percentage of cardiac manifestations and subgroup analyses were calculated based on age and sex.

Results

Data from 38 children who were diagnosed with post-COVID-19 multisystem inflammatory syndrome in children were analyzed. Among these cases, cardiac manifestations were found to be prevalent, specifically relating to valvulopathies such as mitral valve insufficiency (42.1%) and tricuspid valve insufficiency (31.6%). Children over the age of six exhibited a higher prevalence of cardiomegaly. Females had a greater prevalence of mitral valve insufficiency and cardiomegaly, while males showed a higher prevalence of coronary artery dilatation, pericardial effusion, and tricuspid valve insufficiency.

Conclusion

The study emphasizes the frequent occurrence of cardiac manifestations in MIS-C. As this is a single-center study, the findings cannot be generalized. However, it underscores the importance of assessing the disease's impact during the initial presentation, throughout the clinical course, and during the follow-up post-recovery.

## Introduction

Coronavirus disease 2019 (COVID-19) caused by the severe acute respiratory syndrome coronavirus 2 (SARS-CoV-2) was first reported in Wuhan, China, in December 2019 [1,2]. It quickly spread globally, causing the declaration of a pandemic by the World Health Organization (WHO) in March 2020 [3]. Initially, it was believed that children had milder symptoms compared to adults [4,5]. However, in April 2020, reports documented clusters of hospitalized children with severe illness resembling Kawasaki disease and hyperinflammatory shock syndrome [6,7]. On May 14, 2020, the Centers for Disease Control and Prevention (CDC) released an advisory defining a new condition called multisystem inflammatory syndrome in children (MIS-C), characterized by fever, inflammation, and severe illness requiring hospitalization in individuals under 21 years of age, with a temporal association with COVID-19 [8,9]. It often involves dysfunction of multiple organs, including the cardiovascular system.

In Bahrain, COVID-19 cases have been reported across various age groups, including children. From January 2020 to December 2023, the country recorded 696,614 confirmed cases of COVID-19 and 1,536 deaths [10]. With the rising global concern over the prevalence of MIS-C following COVID-19 [11] and the limited national and regional data on the subject, this study seeks to address critical gaps in current knowledge, improve clinical understanding, and provide evidence-based insights. The central research question guiding this study is: What is the prevalence and clinical profile of MIS-C in children within a national context following COVID-19 exposure? Specifically, the study focuses on assessing the prevalence of cardiac manifestations and associated laboratory parameters in children diagnosed with post-COVID-19 MIS-C in the Kingdom of Bahrain.

## Materials and methods

Study setting and population

A retrospective study was conducted at a prominent tertiary healthcare facility in Bahrain, with a total capacity of approximately 1,200 beds, from April 2020 to September 2021. All children admitted with a provisional diagnosis of post-COVID-19 MIS-C were reviewed and screened for inclusion in the study. The diagnosis was made after the disease was suspected clinically with a suggestive history and examination findings when patients presented to the emergency or outpatient department, which were then reviewed by pediatric cardiology consultants. The final diagnosis of post-COVID MIS-C was based on the criteria provided by the WHO [8,12,13] and was confirmed by multiple healthcare providers. A total of 75 patients with characteristics similar to MIS-C were identified, of which 38 met the WHO criteria for post-COVID MIS-C. Patients with a prior diagnosis of Kawasaki disease or other alternative diagnoses were excluded from the study, specifically if they did not meet the WHO diagnostic criteria for post-COVID MIS-C. The distinction between Kawasaki disease was made on the clinical grounds of having a positive COVID-19 swab (or antibodies).

Data collection

Following ethical approval from the Research Committee for Government Hospitals (approval number 50090522), data collection was started. A total of 38 cases were included in the analysis. Patients’ data were obtained from the hospital's medical records, which were documented in a Microsoft Excel sheet (Microsoft Corporation, Redmond, WA, US) and subsequently reviewed by the consultant pediatric cardiologists to determine the final diagnosis, if applicable. Various data were retrieved for analysis, including demographic variables, relevant medical history, presenting symptoms and signs, and duration of fever. Laboratory variables encompassing inflammatory markers, cardiac biomarkers, and other pertinent laboratory findings, and imaging variables encompassing chest X-ray, ECG, echocardiographic, and other cardiac imaging results were collected. Cardiac manifestations included were as follows: clinical (presence of prolonged fever >3 days, systemic edema, gastrointestinal and or upper respiratory symptoms eg. nausea, vomiting, abdominal pain, diarrhea, runny nose or sore throat), radiological (cardiomegaly on chest x-ray, pericardial effusion, mitral valve insufficiency, tricuspid valve insufficiency, left ventricular shortening fraction (LVSF) <28%, z score >2.5), ECG findings (abnormal q waves only), if the patient was ever admitted to the PICU, and laboratory abnormalities as shown of the thresholds in the attached tables (WBC, platelets, CRP, ESR, fibrinogen, procalcitonin, ferritin, d-dimer, B-type natriuretic peptide (BNP), troponin, creatine kinase (CK), lactate dehydrogenase (LDH), interleukin 6 (IL6)). Patients with incomplete or missing data, children with pre-existing cardiac conditions unrelated to MIS-C, and those who had Kawasaki were excluded from the study. All data used in this study were anonymized before analysis. Data collection was completed in December 2021. The data entry was cross-checked after completion, which was done in December 2023.

Data analysis

The data were analyzed using the Statistical Package for the Social Sciences (version 22.0; IBM Corp., Armonk, NY, US). Data were presented using frequencies for categorical data and means and standard deviation (SD) for continuous data. The database where the data analysis was performed did not include any missing values. The prevalence of cardiac manifestation among the studied cases was compared by their age (≤ 6 and > 6 years), and sex using chi-square, Fisher exact, or Mann-Whitney U tests as appropriate. The statistical significance level was set at p < 0.05.

## Results

Thirty-eight children fulfilled the criteria for inclusion in the study as cases of COVID-19 MIS-C at the studied center. Tables 1-2 provide an overview of the demographic, clinical, and laboratory characteristics of pediatric patients diagnosed with post-COVID-19 MIS-C. The mean age of the patients was 6.2 ± 3.6 years, with more than half being male (57.9%, n=22). The majority of patients (81.6%, n=31) had no comorbidities. Fever lasting more than three days was the most common symptom, affecting 68.4% (n=26) of patients. Cardiovascular findings, such as clinically normal cardiac exams (84.2%, n=32) and cardiomegaly on chest X-ray (18.4%, n=7), were observed in some cases. Echocardiographic abnormalities were present in several patients, with mitral valve insufficiency being the most frequent (42.1%, n=16). Laboratory results showed elevated inflammatory markers such as CRP (111.0 ± 63.2 mg/L), ESR (58.1 ± 31.7 mm/h), ferritin (547.9 ± 418.7 ng/L), and D-dimer (4.1 ± 3.8 mg/L). However, mean troponin levels were not elevated. Additionally, a significant proportion of patients required pediatric intensive care unit (PICU) admission (26.3%, n=10), all of whom had cardiac abnormalities in their echocardiographic imaging.

**Table 1:** Demographic and clinical characteristics of pediatric patients with post-COVID-19 MIS-C *Data are presented as n (%) ± SD. Abbreviations: MIS-C, multisystem inflammatory syndrome in children; NAD, no abnormality detected; CXR, chest X-ray; ECG, electrocardiography; LVSF, left ventricular shortening fraction; PICU, pediatric intensive care unit

Characteristics*		n (%)
Demographic characteristics		
Age in years; mean ± SD		6.2 ± 3.6
Age > 6 years		20 (52.6)
Sex		
Male		22 (57.9)
Female		16 (42.1)
Comorbidities		
None		31 (81.6)
Symptoms and signs		
Fever > 3 days		26 (68.4)
Gastrointestinal symptoms**		4 (10.5)
Upper respiratory symptoms**		3 (7.9)
Systemic edema		2 (5.3)
Cardiac examination		
NAD (Clinically)		32 (84.2)
Cardiomegaly (CXR)		7 (18.4)
ECG findings		
Abnormal Q wave		4 (10.5)
Echocardiography features		
Pericardial effusion		5 (13.1)
Mitral valve insufficiency		16 (42.1)
Tricuspid valve insufficiency		12 (31.6)
LVSF <28%		5 (13.2)
Z score > 2.5 (coronary artery dilatation)		4 (10.5)
PICU admission		10 (26.3)

**Table 2:** Laboratory characteristics of pediatric patients with post-COVID-19 MIS-C *Data are presented as mean ± S. Abbreviations: MIS-C, multisystem inflammatory syndrome in children; WBCs, white blood cells; CRP, C-reactive protein; ESR, erythrocyte sedimentation rate; BNP, brain natriuretic peptide; CK, creatinine kinase; LDH, lactate dehydrogenase; IL6, interleukin 6

Laboratory results	Normal range	Result/Finding
WBC (10^3^/µL)	3.6-9.6	10.1± 4.6 ↑
Platelet (10^3^/mL)	150-400	256.9 ± 161.9
CRP (mg/L)	0-3	111.0 ± 63.2 ↑
ESR (mm/h)	<20	58.1 ± 31.7 ↑
Fibrinogen (g/L)	217-496	434.7 ± 148.
Procalcitonin (ng/mL)	0-0.5	27.1 ± 53.1 ↑
Ferritin (ng/L)	16-323	547.9 ± 418.7 ↑
D-dimer (mg/L)	0.09-0.33	4.1 ± 3.8 ↑
BNP (pg/mL)	<21.9	145.0 ± 196.3 ↑
Tropinin (ng/mL)	<1.5	0.03 ± 0.09
CK (U/L)	<228	77.3 ± 110.3
LDH (U/L)	100-300	295.4 ± 80.1
IL6 (pg/mL)	<7	155.2 ± 193.1 ↑

Tables 3-4 compare the cardiac manifestations and laboratory findings in pediatric patients with post-COVID-19 MIS-C, categorized by age group (≤6 years vs. >6 years). Notable differences were observed in cardiac manifestations, such as cardiomegaly (chest X-ray) and abnormal Q waves on ECG, where older children (>6 years) exhibited higher prevalence rates. Laboratory variables also showed age-related differences, with elevated ferritin and troponin levels more common in children over six years, and a higher proportion of older children requiring PICU admission. Statistical analysis revealed significant associations for cardiomegaly (p=0.04), abnormal Q wave (p=0.03), ferritin (p=0.02), troponin (p=0.01), and PICU admission (p=0.04). Other variables, such as WBC count, platelet count, and CRP levels, showed no significant differences between the two age groups.

**Table 3:** Comparison of cardiac variables in pediatric patients with post-COVID-19 MIS-C by age group *Significant; **Analysis done by Fisher's exact test; ***Analysis done by the Mann-Whitney U test Abbreviations: MIS-C, multisystem inflammatory syndrome in children; NAD, no abnormality detected; CXR, chest X-ray; ECG, electrocardiography; LVSF, left ventricular shortening fraction; PICU, pediatric intensive care unit

Cardiac-related variables	Cases ≤ 6 years (n= 18)	Cases > 6 years (n= 20)	Value of test statistic	P value
Cardiac manifestation variables				
Cardiac examination**				
NAD (Clinically)	16 (88.9)	18 (80.0)	1.65	0.54
Cardiomegaly (CXR)	1 (5.6)	6 (30.0)	4.10	0.04*
ECG findings**				
Sinus arrhythmia	1 (5.6)	3 (15.0)	5.06	0.08
Abnormal q wave	0 (0.0)	4 (20.0)	7.02	0.03*
Echocardiography features**				
Pericardial effusion**	2 (16.7)	2 (10.0)	0.59	0.63
Mitral valve insufficiency	6 (33.3)	10 (50.0)	1.02	0.29
Tricuspid valve insufficiency	4 (22.2)	8 (40.0)	1.16	0.30
LVSF <28%	2 (11.1)	3 (15.0)	0.34	0.52
Z score > 2.5 (coronary artery dilatation)	2 (11.1)	2 (10.0)	0.11	0.78
PICU admission**	2 (11.2)	8 (40.0)	2.20	0.04*

**Table 4:** Comparison of laboratory variables in pediatric patients with post-COVID-19 MIS-C by age group *Significant; **Analysis done by Fisher's exact test; ***Analysis done by the Mann-Whitney U test Abbreviations: MIS-C, multisystem inflammatory syndrome in children; WBCs, white blood cells; CRP, C-reactive protein; ESR, erythrocyte sedimentation rate; BNP, brain natriuretic peptide; CK, creatinine kinase; LDH, lactate dehydrogenase; IL6, interleukin 6

Laboratory-related variables	Cases ≤ 6 years (n= 18)	Cases > 6 years (n= 20)	Value of test statistic	P value
WBCs (10^3^/µL)***	9.7 ± 4.5	10.2 ± 4.8	191.0	0.75
Platelets (10^3^/mL)***	222.3 ± 128.7	288.2 ± 184.6	223.5	0.20
Elevated CRP (mg/L)**	18 (100.0)	19 (95.0)	0.07	1.00
Elevated ESR (mm/h)**	17 (94.4)	19 (95.0)	0.95	1.00
Elevated Fibrinogen (g/L)**	9 (50.0)	16 (80.0)	1.92	0.08
Elevated Procalcitonin (ng/mL)	6 (33.3)	9 (45.0)	0.72	0.51
Elevated Ferritin (ng/L)**	10 (55.8)	18 (90.0)	2.40	0.01*
Elevated D-dimer mg/L)**	11 (61.1)	12 (60.0)	0.07	1.00
Elevated BNP (pg/mL)**	10 (55.6)	14 (70.0)	0.90	0.50
Elevated Tropinin (ng/mL)**	0 (0.0)	6 (30.0)	2.49	0.01*
Elevated CK (U/L)**	2 (11.1)	3 (15.0)	0.35	1.00
Elevated LDH (U/L)**	10 (55.6)	8 (40.0)	0.94	0.51
Elevated IL6 (pg/mL)**	14 (77.6)	14 (70.0)	0.54	0.71

Tables 5-6 compare the cardiac manifestations and laboratory findings between male and female pediatric patients diagnosed with post-COVID-19 MIS-C. Despite the small sample size, the analysis shows no significant gender-based differences in most cardiac manifestations, including clinical examination findings, cardiomegaly, and echocardiographic features such as mitral valve insufficiency and tricuspid valve insufficiency. However, coronary artery dilatation (Z score > 2.5) was more prevalent in males (13.6%, n=3) as compared to females (6.3%, n=1) with a significant difference (p=0.04). Regarding laboratory results, both sexes exhibited similar patterns, with no significant differences in inflammatory markers, including CRP, ESR, and ferritin. The rate of PICU admission was also similar between males (27.3%, n=6) and females (25.0%, n=4), indicating no gender-specific trends in critical care needs.

**Table 5:** Comparison of cardiac variables between male and female pediatric patients with post-COVID-19 MIS-C *Significant; **Analysis done by Fisher's exact test; ***Analysis done by the Mann-Whitney U test Abbreviations: MIS-C, multisystem inflammatory syndrome in children; ECG, electrocardiography; LVSF, left ventricular shortening fraction; PICU, pediatric intensive care unit

Cardiac-related variables		Male cases (n= 22)	Female cases (n= 16)	Value test statistic	P value
Cardiac manifestation variables					
Cardiac examination**					
No abnormality detected (Clinically)		19 (86.4)	13 (81.3)	0.40	0.91
Cardiomegaly (chest radiograph)		3 (13.6)	4 (25.0)	0.88	0.42
ECG findings**					
Abnormal q wave		2 (9.1)	2 (12.5)	0.33	1.00
Echocardiography features**					
Pericardial effusion		3 (13.6)	2 (12.5)	0.10	1.00
Mitral valve insufficiency		8 (36.4)	8 (50.0)	0.68	0.40
Tricuspid valve insufficiency		8 (36.4)	4 (25.0)	0.73	0.50
LVSF <28%		2 (9.1)	3 (18.8)	0.86	0.63
Z score > 2.5 (coronary artery dilatation)		3 (13.6)	1 (6.3)	0.72	0.62
PICU admission**		6 (27.3)	4 (25.0)	0.29	0.98

**Table 6:** Comparison of laboratory variables between male and female pediatric patients with post-COVID-19 MIS-C *Significant; **Analysis done by Fisher's exact test; ***Analysis done by the Mann-Whitney U test Abbreviations: MIS-C, multisystem inflammatory syndrome in children; WBCs, white blood cells; CRP, C-reactive protein; ESR, erythrocyte sedimentation rate; BNP, brain natriuretic peptide; CK, creatinine kinase; LDH, lactate dehydrogenase; IL6, interleukin 6

Laboratory-related variables		Male cases (n=22)	Female cases (n=16)	Value test statistic	P value
WBC (10^3^/µL)***	Normal 3.6-9.6	10.6 ± 4.4	9.02 ± 4.9	139.5	0.28
Platelet (10^3^/mL)***	Normal 150-400	254.8 ± 138.1	259.9 ± 197.8	152.5	0.48
CRP (mg/L) **	Normal 0-3	21 (95.5)	15 (93.8)	0.22	1.00
ESR (mm/h) **	Normal <20	22 (100.0)	15 (93.4)	1.17	0.42
Fibrinogen (g/L) **	Normal 217-496	15 (68.2)	10 (62.5)	0.36	0.71
Procalcitonin (ng/mL) **	Normal 0-0.5	7 (31.8)	8 (50.0)	1.11	0.25
Ferritin (ng/L) **	Normal 16-323	16 (72.7)	12 (75.0)	0.15	1.00
D-dimer mg/L) **	Normal 0.09-0.33	14 (63.6)	9 (56.3)	0.45	0.74
BNP (pg/mL)	Normal <21.9	14 (63.6)	10 (62.5)	0.07	0.94
Tropinin (ng/mL) **	Normal <1.5	4 (18.2)	2 (12.5)	0.22	0.68
CK (U/L) **	Normal <228	3 (13.6)	2 (12.5)	0.10	1.00
LDH (U/L) **	Normal 100-300	11 (50.0)	7 (45.8)	0.37	0.70
IL6 (pg/mL) **	Normal <7	16 (72.7)	12 (75.0)	0.15	1.00

## Discussion

This study aimed to assess the prevalence and clinical characteristics of cardiac manifestations in children diagnosed with post-COVID-19 MIS-C at a tertiary hospital in Bahrain. Among the studied children, the majority (68.4%, n=26) presented with a fever lasting more than three days, a common symptom associated with this condition. Gastrointestinal symptoms were observed in 10.5% (n=4) of the cases, while 7.9% (n=3) had upper respiratory symptoms, suggesting that MIS-C can present with various clinical presentations. Similar findings were also reported from a recent report on 15 patients from another tertiary center in Bahrain, where prolonged fever, gastrointestinal, respiratory, and eye symptoms were the main clinical presentations in these patients [14]. Other studies also found that fever and gastrointestinal symptoms, including abdominal pain, vomiting, and/or diarrhea, are the most common manifestations [11,15].

In terms of cardiac involvement, the majority (81.6%, n=31) had negative findings during clinical examination, but investigations such as chest X-ray, ECG, echocardiography, and laboratory tests revealed significant manifestations. The most common echocardiographic finding was valvular insufficiency; specifically, mitral valve and tricuspid valve insufficiency. Stratified analyses revealed a higher prevalence of mitral valve insufficiency in older children (≥6 years) at 50% (n=10), compared to 33.3% (n=6) in those under 6 years. Additionally, it was more common in females (50%, n=8) than in males (36.4%, n=8). Tricuspid insufficiency, however, was more prevalent in males and older patients. Previous studies have reported similar findings, demonstrating a significant proportion of patients had valvular insufficiency and ventricular dysfunction [15,16]. Arrhythmias and conduction abnormalities were found to be common in patients with MIS-C [17,18]. Our study detected an abnormal Q wave in 20% of older patients. Consistent with these findings, Regan et al. reported that the most common ECG findings were low QRS amplitude and Q and T wave abnormalities [18]. In another report, first-degree heart block was observed among MIS-C patients [19]. Pericardial involvement does not necessarily accompany myocardial involvement, but mild-to-moderate pericardial effusion or even severe effusion with tamponade have been reported [20,21]. In our study, pericardial effusion was observed in only 5 cases (13.1%). Similarly, dilated coronary arteries (z-score greater than 2.5) were found in 4 cases (10.5%), with a significantly higher prevalence in males (13.6%, n=3). Previous studies, however, have reported varying degrees and rates of coronary artery involvement among MIS-C cases [7,22,23]. Several studies have reported a high prevalence of decreased left ventricular shortening fraction (LVSF) [16,22,23]. In our study, a reduced LVSF was observed in 13.2% (n=5) of the cases, with a higher frequency in female patients (18.8%, n=3) and older children (15%, n=3). The exact underlying mechanisms of these cardiac abnormalities are still unclear, and further longitudinal studies are needed to shed light on the pathophysiology of these cardiac manifestations. However, some reports suggest that myocardial damage may occur due to direct viral injury to the myocardium and the hyperinflammatory state [24,25]. In cases where cardiac involvement is suspected, laboratory findings can provide valuable insights. In our study, we observed elevated mean BNP concentrations, indicating potential cardiac involvement, particularly in older children (70%, n=14) and males (63.6%, n=14). However, mean levels of CK, troponin, and LDH remained within the normal range, suggesting that cardiac injury may not be severe or widespread; however, when elevated in individual cases, they still retain their clinical significance. It is worth noting that elevated cardiac enzymes can indicate severe cardiac injury [17]. Moreover, significantly elevated levels of IL6 were observed in all the children, especially in females (75%, n=12) and younger children (77.6%, n=14), with an estimated average of 155.2. Additionally, several other laboratory findings, including D-dimer, ferritin, fibrinogen, procalcitonin, ESR, and CRP, were elevated. These findings indicate a strong inflammatory status, coagulation abnormalities, and systemic involvement in MIS-C [26]. Therefore, during the COVID-19 pandemic, it was important to consider MIS-C as a potential cause in the differential diagnosis of severe systemic inflammatory or infectious conditions such as Kawasaki disease. Both are differentiated based on: 1. age (Kawasaki disease is seen in younger patients, and MIS-C occurs frequently in adolescents and teens), 2. Presence of an epidemiological link to COVID (history of infection or exposure to a case, positive PCR or serology), 3. Coronary artery involvement is more common in Kawasaki disease. At the same time, high levels of cardiac enzymes, ventricular dysfunction, and hemodynamic instability are associated with MIS-C, as well as gastrointestinal and respiratory symptoms. 4. Labs (MIS-C is more commonly associated with thrombocytopenia and elevated d-dimer and ferritin, while Kawasaki is associated with thrombocytosis, leukocytosis, and eosinophilia) [27].

The study findings revealed that approximately 26.3% (n=10) of the children required admission to the PICU. This was particularly significant among males and children older than six years, suggesting a higher severity of the condition in certain cases. In contrast, higher rates of PICU admissions have been reported [28], and this disparity may be attributed to different variants of the SARS-CoV-2 virus, which could influence the inflammatory response based on the virus genotype [29]. Initially, MIS-C was compared to Kawasaki disease, but our study highlights an important epidemiological difference in terms of patient age. MIS-C appears to be more prevalent and severe in children older than six years, which can aid clinicians in distinguishing between these two conditions.

In this study, selection bias was tackled by using specific WHO criteria to select cases, which rely on objective clinical findings, as well as including all patients who presented to the outpatient and emergency departments in the tertiary center where the study was performed. The possible variability in diagnostic assessments (two cardiologists) was also addressed during the clinical course of the disease, as all the patients were seen physically and their diagnosis and treatment plans were regularly revised and discussed by both cardiologists.

## Conclusions

Although our study has enrolled a relatively large number of patients for a single, tertiary-care center, it is important to consider the relatively limited sample size when it comes to generalizing the findings. The lack of long-term follow-up of the patients after the period when the study was completed is another limitation, emphasizing the need for larger multicenter cohort studies, collaborative efforts across multiple centers to strengthen the validity of the conclusions, and a deeper look into myocardial recovery as MIS-C is a novel disease and is not well studied.

In conclusion, this study provides insights into the cardiac manifestations and clinical features of post-COVID-19 MIS-C in pediatric patients in Bahrain. It highlights that older children (≥6 years) and males were more likely to experience cardiac abnormalities, such as coronary artery dilatation, and required more intensive care. Elevated inflammatory markers were common across patients, suggesting an inflammatory etiology for the said cardiac features. The findings emphasize the need for early detection and targeted management, especially in higher-risk groups, and suggest the importance of further research to understand long-term outcomes and the impact of different SARS-CoV-2 variants. On a clinical note, the study highlights the need for routine cardiac screening in at-risk patients presenting with MIS-C.
